# GATA3 protein as a *MUC1 *transcriptional regulator in breast cancer cells

**DOI:** 10.1186/bcr1617

**Published:** 2006-11-01

**Authors:** Martín C Abba, María I Nunez, Andrea G Colussi, María V Croce, Amada Segal-Eiras, C Marcelo Aldaz

**Affiliations:** 1 Department of Carcinogenesis, The University of Texas MD Anderson Cancer Center, Science Park – Research Division, PO Box 389, Smithville, TX 78957, USA; 2 Centro de Investigaciones Inmunológicas Básicas y Aplicadas (CINIBA), Facultad de Ciencias Médicas, Universidad Nacional de La Plata, Calle 60 y 120, La Plata, CP:1900, Argentina

## Abstract

**Introduction:**

Recent studies have demonstrated that members of the GATA-binding protein (GATA) family (GATA4 and GATA5) might have pivotal roles in the transcriptional upregulation of mucin genes (*MUC2*, *MUC3 *and *MUC4*) in gastrointestinal epithelium. The zinc-finger GATA3 transcription factor has been reported to be involved in the growth control and differentiation of breast epithelial cells. In SAGE (serial analysis of gene expression) studies we observed an intriguing significant correlation between *GATA3 *and *MUC1 *mRNA expression in breast carcinomas. We therefore designed the present study to elucidate whether *MUC1 *expression is regulated by GATA3 in breast cancer cells.

**Methods:**

Promoter sequence analysis of the *MUC1 *gene identified six GATA *cis *consensus elements in the 5' flanking region (GATA1, GATA3 and four GATA-like sequences). Chromatin immunoprecipitation and electrophoretic mobility-shift assays were employed to study the presence of a functional GATA3-binding site. *GATA3 *and *MUC1 *expression was analyzed *in vitro *with a GATA3 knockdown assay. Furthermore, expression of *GATA3 *and *MUC1 *genes was analyzed by real-time RT-PCR and immunohistochemistry on breast cancer-specific tissue microarrays.

**Results:**

We confirmed the presence of a functional GATA3-binding site on the *MUC1 *promoter region in the MCF7 cell line. We determined that GATA3 knockdown assays led to a decrease in MUC1 protein expression in MCF7 and T47D cells. In addition, we detected a statistically significant correlation in expression between *GATA3 *and *MUC1 *genes at the mRNA and protein levels both in normal breast epithelium and in breast carcinomas (*p *= 0.01). GATA3 expression was also highly associated with estrogen receptor and progesterone receptor status (*p *= 0.0001) and tumor grade (*p *= 0.004) in breast carcinomas.

**Conclusion:**

Our study provides evidence indicating that GATA3 is probably a mediator for the transcriptional upregulation of *MUC1 *expression in some breast cancers.

## Introduction

GATA3 (GATA-binding protein 3) belongs to a family of transcription factors (GATA1 to GATA6) that bind with high affinity to the consensus sequence (A/T)GATA(A/G) and share a steroid-hormone-receptor superfamily C4 zinc-finger DNA-binding motif [1]. GATA factors are classified into two subfamilies on the basis of structural features and expression patterns. The expression of *GATA1*, *GATA2*, and *GATA3 *has been detected predominantly in hematopoietic cells, whereas *GATA4*, *GATA5*, and *GATA6 *are expressed mainly in the cardiovascular system and in endodermal-derived tissues including liver, lung, pancreas, and intestine [2]. The function of GATA factors is modulated by their interaction with other transcription factors, transcriptional coactivators and co-repressors.

In genome-wide expression profile studies from our laboratory we observed that the expression of *GATA3 *is highly correlated with estrogen receptor-α (ERα) status in breast carcinomas [3] similar results were reported by others [4-9]. Parikh and colleagues (2005) suggested that GATA3 expression might be associated with responsiveness to hormone therapy in breast cancer patients [10]. Furthermore, the expression of *GATA3 *has been shown to correlate with specific breast cancer phenotypes, defined as luminal type A, carrying an improved disease-free survival and overall survival when compared with tumors that do not express *GATA3 *[11]. It has been reported that GATA3 may be involved in growth control and differentiation in breast epithelial cells mediating the transcriptional activation of several genes such as those encoding cytokeratins 5, 6 and 17, and trefoil factors 1 and 3 [12]. Recent evidence indicates that the proteins GATA4, GATA5, and GATA6 may be important in the upregulation of mucin expression (*MUC2*, *MUC3*, and *MUC4*) and trefoil factor genes (*TFF1 *and *TFF2*), events that are in turn associated with gastrointestinal epithelial cell differentiation [13-15].

The MUC1 glycoprotein is a member of the mucin family of proteins, expressed mostly on the apical membrane of various glandular epithelia such as in luminal breast epithelial cells [16]. The association of *MUC1 *overexpression with loss of cell polarity has been observed in breast carcinomas. Abnormal *MUC1 *expression leads to a loss of cell–cell and cell–extracellular-matrix adhesion [17]. It was further determined that this increase in *MUC1 *expression is due mainly to transcriptional regulatory events [18].

The 5'-regulatory region of the human *MUC1 *gene was analyzed previously [19-21]. Several consensus binding sites for transcription factors were observed in this promoter region, such as those for the SP1, STAT1, STAT3, NF-κB, MZF1 and DbpA proteins, all of which may be involved in the transcriptional regulation of *MUC1 *[18,20,22]. However, the factors determining *MUC1 *tissue-specific expression remain largely unknown, as do the mechanisms causing *MUC1 *overexpression in tumors.

Global gene expression studies pointed to a significant correlation in the overexpression of *GATA3 *and *MUC1 *genes commonly observed in breast cancer. Interestingly, we also observed the presence of multiple putative GATA-binding sites throughout the *MUC1 *promoter. Thus, the aim of this study was to evaluate the role of GATA3 as a putative transcriptional regulator of *MUC1 *in breast cancer.

## Materials and methods

### Serial analysis of gene expression database mining

To perform a comparative analysis of the GATA family members expressed in breast tissue, we analyzed 47 breast SAGE (serial analysis of gene expression) libraries: 4 normal breast epithelium, 8 ductal carcinoma *in situ *(DCIS), 33 invasive ductal carcinomas (IDCs), and the MCF7 and ZR75 breast cancer cell lines. To this end, we combined 29 breast cancer SAGE libraries generated by us at a resolution of 100,000 tags per library (Aldaz Laboratory) with 18 SAGE libraries (generated at the Polyak Laboratory, Dana-Farber Cancer Institute, Boston, MA, USA) downloaded from the Cancer Genome Anatomy Project – SAGE Genie database [23]. SAGE data management and tag-to-gene matching for *GATA1 *(GCCTCCAGAG), *GATA2 *(GACAGTTGTT), *GATA3 *(AAGGATGCCA), *GATA4 *(TCTCTCCCCT), *GATA5 *(TCCTGGCATA), *GATA6 *(GAGAAGATCA), *ESR1 *(AGCAGGTGCC), and *MUC1 *(CCTGGGAAGT) were performed with a suite of web-based SAGE library annotation tools developed by us [24]. To enable the visualization and illustration of our analyses, we used the TIGR MultiExperiment Viewer (MeV 2.2) software (The Institute for Genomic Research, Rockville, MD, USA). This tool was employed for normalization and average clustering of the SAGE data. Spearman's test was employed for a correlation analysis of transcripts. The CGAP-dbEST and Oncomine databases [25] were employed for collection and visualization of gene expression profiles of the previously mentioned transcripts from publicly available breast cancer ESTs and microarray data sets (Additional file 1).

### Real-time RT-PCR expression analysis in breast carcinomas

For validation studies, total RNA was isolated from an independent set of 36 snap-frozen breast carcinomas (stages I and II: 13 ERα-negative tumors and 23 ERα-positive tumors) using TRIzol reagent (Invitrogen, San Francisco, CA, USA). Template cDNAs were synthesized with the SuperScript™ First-strand Synthesis System (Invitrogen). *GATA3 *and *MUC1 *primers and probes were obtained from Applied Biosystems (TaqMan Assays-on-Demand™ Gene Expression Products; Foster City, CA, USA). All the PCR reactions were performed with the TaqMan PCR Core Reagents kit and the ABI Prism^® ^7700 Sequence Detection System (Applied Biosystems). Experiments were performed in duplicate for each data point and 18S rRNA was used as control. Results are expressed as means ± 2 SEM based on log_2 _transformation of normalized real-time RT-PCR values of the assayed genes.

### Tissue microarray and immunostaining analysis

We used breast-specific tissue microarrays (TMAs) from two sources (Fox Chase Cancer Center, Philadelphia, and Cooperative Breast Cancer Tissue Resource, NCI-CBCTR) for GATA3 and MUC1 protein expression profiling. We analyzed a total of 263 cases (38 normal tissues (19 DCIS and 206 IDCs). Information on ER and progesterone receptor (PR) status was available for all IDC cases. Before immunostaining, endogenous peroxidase activity was blocked with 3% H_2_O_2 _in water for 10 minutes. Heat-induced epitope retrieve was performed with 1.0 mM EDTA buffer pH 8.0 for 10 minutes in a microwave oven followed by cooling for 20 minutes. To block non-specific antibody binding, the slides were incubated with 10% goat serum in PBS for 30 minutes. Primary monoclonal anti-GATA3 (HG3-31: SC-268; Santa Cruz Biotechnology, Santa Cruz, CA, USA) and anti-MUC1 (VU4H5: SC-7313; Santa Cruz Biotechnology) antibodies were used at 1:50 and 1:100 dilutions respectively. Antibody detection was performed using diaminobenzidine (DAB). Staining intensity was determined by means of a Chromavision Automated Cellular Imaging System (ACIS^®^) with the generic DAB software application, as described previously [26]. This software determines brown intensity regardless of the area covered by the positive cells.

Multivariate analysis was performed by principal components analysis (PCA). Variables were codified and transformed as follows: negative staining (0) and positive staining (1) for ER, PR, *GATA3 *and *MUC1 *expression; normal tissue (0), DCIS (1) and IDC (2) for histology; lymph node-negative (0) and lymph node-positive (1) status; and low (1), moderate (2) and high (3) tumor grade. To enable visualization of the factorial analysis, we employed a three-dimensional representation of component plot in rotated space. The basic significance level was fixed at *p *< 0.05 and all data were analyzed with SPSS^® ^statistical software (SPSS Inc., Chicago, IL, USA).

### Cell culture, western blot and immunocytochemistry analyses

The estrogen-dependent breast cancer cell lines MCF7 and T47D were grown in RPMI-1640 medium (Gibco, Gaithersburg, MD, USA) supplemented with 10% (v/v) FBS (Bioser, Buenos Aires, Argentina), 10 U/ml penicillin G, and 10 μg/ml streptomycin.

Trypan blue staining was used to assess cell viability. Cells (3 × 10^6^) were lysed with 1 ml of RIPA buffer (50 mM Tris pH 7.5, 150 mM NaCl, 0.5% sodium deoxycholate, 1% Triton X-100, 0.1% SDS) containing protease inhibitor. For western blotting, 50 μl of cell extract was separated by 10% SDS-PAGE and transferred to nitrocellulose membrane (Protran^®^; BioScience, Dassel, Germany). Immunodetection was performed with a GATA3 mouse monoclonal antibody (HG3-31) at 1:300 dilution, followed by incubation with horseradish peroxidase (HRP)-conjugated secondary antibody (anti-mouse-HRP; Sigma, St Louis, MO, USA) at 1:1000 dilution. Immunostaining was performed with DAB as substrate.

MCF7 cells were also grown on coverslips; these preparations were washed twice with 1 × PBS and fixed with 10% formaldehyde in 1 × PBS. Immunodetection of GATA3 was assessed with HG3-31 monoclonal antibody. Primary antibodies were detected with biotin-conjugated goat anti-mouse IgG, followed by incubation with streptavidin-conjugated HRP and using DAB as a substrate. The cells grown on coverslips were counterstained with hematoxylin and examined by light microscopy.

### *MUC1 *promoter sequence analysis and chromatin immunoprecipitation

The complete sequence of the human *MUC1 *promoter region was obtained from GenBank (accession no. NM_002456). We used the DNAMAN software (Version 4.15; Lynnon BioSoft, Vaudreuil-Dorion, Quebec, Canada) to identify putative GATA-binding sites (A/T-GATA-A/G) in this regulatory sequence.

MCF7 breast cancer cells were grown to 90% confluence; culture medium was then removed. Cells were washed and fixed with 1% formaldehyde in 1 × PBS for 10 minutes at room temperature. Fixed cells were washed twice with 1 × PBS, scraped off and incubated in 1 ml of lysis buffer A (20 mM HEPES pH 7.9, 25% glycerol, 420 mM NaCl, 1.5 mM MgCl_2_, 0.2 mM EDTA, 1 mM phenylmethylsulphonyl fluoride), followed by a second incubation in 300 μl of lysis buffer B (50 mM Tris–HCl pH 8.0, 1 mM EDTA, 150 mM NaCl, 1% SDS, 2% Triton X-100). The cell lysate was then sonicated with a Branson Sonifier 450 (30-second pulses at 40% output) and subjected to immunoprecipitation with mouse monoclonal antibody HG3-31 against GATA3, at 4°C for 1 hour; 50 μl of Protein A–Sepharose (1:1 slurry) was added to the reaction and incubated at 4°C for 4 hours. The unbound proteins were removed by washing the Protein A–Sepharose with Triton buffer (50 mM Tris–HCl pH 8.0, 1 mM EDTA, 150 mM NaCl, 0.1% Triton X-100) and 1 × PBS. The antigen–antibody complex was eluted with SDS-NaCl-dithiothreitol (DTT) buffer (62.5 mM Tris–HCl pH 6.8, 200 mM NaCl, 2% SDS, 10 mM DTT). The eluted protein–DNA complex was incubated overnight at 68°C. DNA was isolated with the phenol/chloroform extraction and ethanol precipitation protocol.

The PCR reactions were performed with 15 ng of DNA, 2.5 mM MgCl_2_, each dNTP at 200 μM, 25 pmol of *muc1p1 *forward primer (5'-tagaagggtggggctattcc-3'), 25 pmol of *muc1p1 *reverse primer (5'-taggtcgaggtcctgtacag-3', flanking the GATA1-binding site), 25 pmol of *muc1p2 *forward primer (5'-tttggctgatttggggatgc-3'), 25 pmol of *muc1p2 *reverse primer (5'-aatattgcactcgtcccgtc-3', flanking the GATA3-binding site), and 1.25 units of *Taq *DNA polymerase (Promega, Madison, WI, USA), in PCR buffer (20 mM Tris–HCl pH 8.4, 50 mM KCl) in a final volume of 50 μl. The reactions were cycled as follows: 1 cycle of 94°C for 2 minutes and 25 cycles of 94°C for 1 minute, 56°C for 1 minute, and 72°C for 1 minute. HLA-DQα 1 amplicon (242 bp) was used as control (exon sequence without a GATA-binding site) [27]. Separation and detection of the amplified fragments were performed by electrophoresis on a 6% minigel (19:1 polyacrylamide:bisacrylamide) and staining with silver.

### Electrophoretic mobility-shift assay

Nuclear protein extract from the MCF7 cell line was prepared as described [28]. The following double-stranded DNA probes (27 bp) were used: the wild-type *GATA *consensus oligonucleotide (5'-cacttgataacagaaagtgataactct-3') as positive control [4]; the *MUC1 *promoter sequence encoding the putative GATA3-binding site (5'-ggcggatctttgatagactggagtgtc-3'); and a double GATA3-binding site mutated variant (with transitions G→C and A→T: 5'-ggcggatctttcttagactggagtgtc-3'). A non-isotopic electrophoretic mobility-shift assay (EMSA) was performed with 5 μg of nuclear cell extracts and 50 ng of the probe, 1 μg of salmon testes DNA (Sigma-Aldrich, St Louis, MO, USA) in a 5 × binding reaction buffer (100 mM Hepes pH 7.9, 250 mM KCl, 2.5 mM DTT, 0.25 mM EDTA, 5 mM MgCl_2_, 25% glycerol). After 20 minutes the samples were separated on a 10% polyacrylamide (39:1 polyacrylamide:bisacrylamide) at a constant temperature of 4°C with 1 × Tris-borate-EDTA (45 mA for 4.5 hours). Gels were stained with a previously described silver staining technique [27]. Similarly, for the supershift assay, 1 μg of specific antibody (anti-GATA3) or control antibody (anti-ERβ) was incubated for 20 minutes in the MCF7 nuclear protein extract before the addition of probes. Samples were size separated by electrophoresis in a 10% polyacrylamide minigel (39:1 polyacrylamide:bisacrylamide) at room temperature with 0.5 × Tris-borate-EDTA.

### *GATA3 *anti-sense phosphorothioate oligonucleotide assay

Anti-sense oligodeoxynucleotides were synthesized that matched the translational start region of *GATA3 *(5'-cgccgtcacctccatggcctc-3') [29]. The *GATA3 *anti-sense oligodeoxynucleotide used was synthesized on a phosphorothioate backbone to improve resistance to endonucleases (IDT, Coralville, IA, USA). MCF7 and T47D cells were plated on 50 mm cultured dishes at 50% confluence in Opti-MEM^® ^I Reduced Serum Medium (Invitrogen) and transiently transfected with 3 μg of *GATA3 *anti-sense mixed with Lipofectamine in accordance with the manufacturer's protocol (Invitrogen). Cells were maintained at 37°C and harvested at two time points after transfection (48 and 72 hours). Cells were lysed in RIPA buffer, and total protein concentration was estimated by Lowry's method. Equal amounts of protein were used for the western blot analyses. GATA3 and MUC1 immunodetection were assessed with HG3-31 and CT2 (epitope corresponding to the carboxy terminus of MUC1) monoclonal antibodies, respectively. Primary antibodies were detected with biotin-conjugated goat anti-mouse IgG (for HG3-31) or biotin-SP-conjugated goat anti-Armenian hamster IgG (for CT2) (Jackson ImmunoResearch), followed by incubation with streptavidin-conjugated HRP and using DAB as a substrate. SDS-PAGE with silver staining was employed for the detection of total protein loaded.

In addition, we analyzed the expression of MUC1 protein and its response to *GATA3 *anti-sense by an ELISA assay with MCF7 cell culture. In brief, MCF7 cells were cultured on a 96-well microtitre plate and treated with 0.05 μg of *GATA3 *anti-sense (48 hours), and then blocked with 1% BSA. MUC1 immunodetection was performed with HMFG1 monoclonal antibody. The bound primary antibody was detected with peroxidase-conjugated goat anti-mouse IgG. Color was developed with ABTS (2,2'-Azinobis (3-ethylbenzthiazoline-6-sulfonic acid)) substrate solution and absorbance was measured at 405 nm with a microplate reader (SLT Spectra, SLT Labinstruments, Salzburg, Austria). *MUC1 *expression was expressed as mean optical density with a 95% confidence interval. Statistically significant difference was estimated by *t *test (*p *< 0.05).

## Results

### GATA transcription factors family members expressed in breast epithelial cells

**Figure 1:**
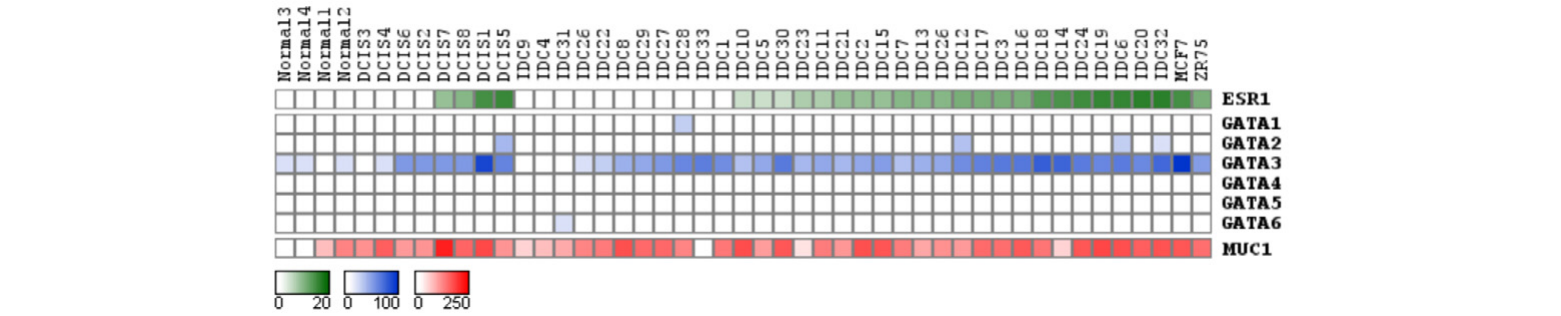
Digital northern analysis based on SAGE data of *GATA *family members, *ESR1 *and *MUC1 *genes in 47 breast SAGE libraries. The color scale at the bottom represents expression level based on numbers of transcripts (tags) per library. SAGE, serial analysis of gene expression.

Interestingly, among the *GATA *family members *GATA3 *was by far the most frequently expressed *GATA *transcription factor in breast cancer. Specifically, *GATA3 *was detected as being expressed in 90% of the breast cancer cases (42 out of 47) according to SAGE database analyses. In contrast, *GATA *family members *GATA1*, *GATA2*, *GATA4*, *GATA5*, and *GATA6 *were detected as expressed in only 13% (6 out of 47) of the SAGE libraries (Figure 1). Similarly, expressed sequence tag database and DNA microarray analyses showed that the *GATA3 *gene is expressed mainly in breast carcinomas compared with other *GATA *genes (Additional file 1). The MCF7 breast cancer cell line displayed the highest *GATA3 *expression level observed. We detected a significant positive correlation between *ESR1 *(the gene encoding ERα) and *GATA3 *tags (*r *= 0.702; *p *= 0.0001), between *ESR1 *and *MUC1 *(*r *= 0.46; *p *= 0.001), and between *GATA3 *and *MUC1 *(*r *= 0.45; *p *= 0.002) (Figure 1).

### *GATA3 *and *MUC1 *real-time RT-PCR analysis in breast carcinomas

**Figure 2:**
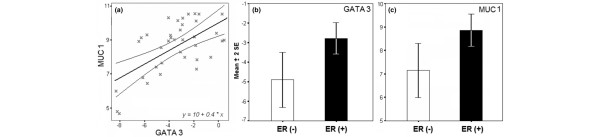
*GATA3 *and *MUC1 *real-time RT-PCR analysis in 36 invasive breast carcinomas. **(a) **Linear regression analysis of *GATA3 *and *MUC1 *mRNA expression levels in invasive ductal carcinoma (IDC) with 95% mean prediction interval (*r *= 0.6; *p *< 0.0001). **(b,c) ***GATA3 *(*p *= 0.008) (b) and *MUC1 *(*p *= 0.009) (c) mRNA expression of IDC in association with ERα status. Results are real-time RT-PCR values of the assayed gene relative to 18S rRNA used as normalizing control and are shown as means ± 2 SEM based on a log_2 _transformation.

To validate the relationship of *GATA3 *and *MUC1 *in breast cancer epithelial cell further, we performed an mRNA expression analysis of 36 primary breast carcinomas by means of real-time RT-PCR. These data showed a significant positive correlation between the mRNA levels of both markers (*r *= 0.6; *p *< 0.0001) (Figure 2a). Furthermore, mRNA expression of *GATA3 *and *MUC1 *correlated positively with breast carcinomas ERα status (*r *= 0.492, *p *= 0.004; *r *= 0.608, *p *= 0.001, respectively) (Figure 2b,c).

### GATA3 and MUC1 tissue-microarray analysis

**Figure 3:**
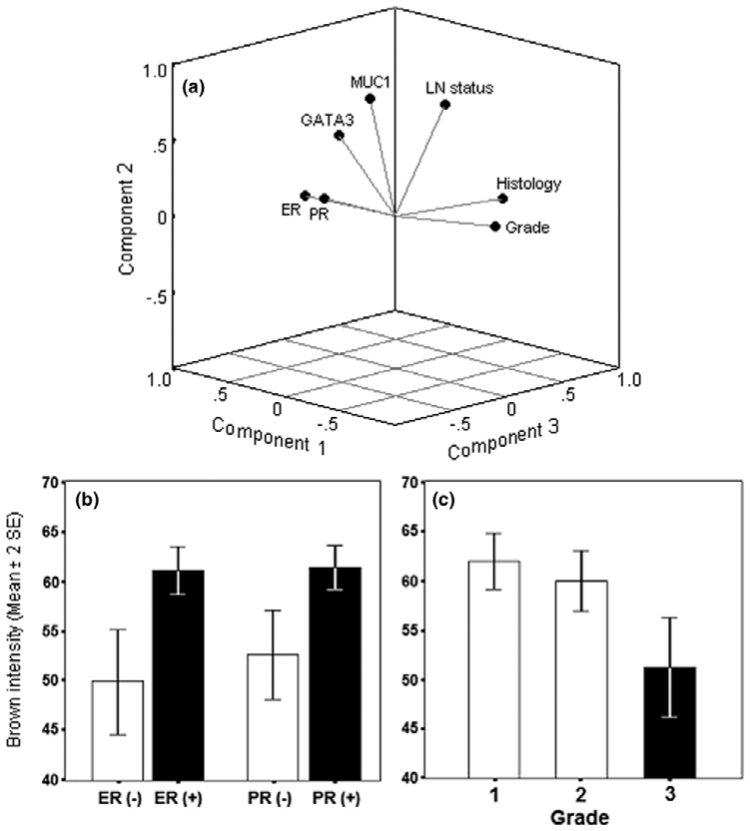
Tissue microarray analysis of 263 breast normal and cancer tissue samples. **(a) **Component plot in rotated space showing factors positively correlated, namely estrogen receptor/progesterone receptor (ER/PR; *p *= 0.0001), ER–PR/GATA3 (*p *= 0.001), and MUC1/lymph node status (*p *= 0.006), GATA3/MUC1 (*p *= 0.01); and factors negatively correlated, namely ER–PR/tumor grade (*p *= 0.0001) and GATA3/tumor grade (*p *= 0.002). **(b,c) **Univariate analysis showing highly statistical association between GATA3 immunostaining and ER and PR status (*p *= 0.0001) (b) and statistical decrease trend in GATA3 immunostaining and the tumor grade of invasive component (*p *= 0.004) (c). Results are based on the brown intensity of immunostaining as described in the Materials and methods section, and are shown as means ± 2 SEM.

PCA of 263 breast tissue samples (Figure 3a; Additional file 2) identified statistically significant positive correlations between *GATA3 *expression and ER/PR status (τ_b _= 0.53; *p *= 0.001) (Figure 3a,b). It is interesting to note that GATA3 and MUC1 protein expression are positively correlated among normal breast samples as well as in breast carcinomas (τ_b _= 0.44; *p *= 0.01). Additionally, *MUC1 *expression was correlated with lymph node-positive status in breast carcinomas (τ_b _= 0.46; *p *= 0.006). We detected a strong correlation between the lack of *GATA3 *expression and higher histological tumor grade (poorly differentiated) demonstrated by multivariate (τ_b _= -0.49; *p *= 0.002) and univariate (*p *= 0.004) analyses (Figure 3a,c). Whereas 54.3% (19 out of 35) of IDC grade I and 45% (40 out of 89) of IDC grade II tumors were GATA3-positive, only 27% (18 out of 67) of IDC grade III tumors were GATA3-positive (*p *= 0.013).

Overall, these studies showed a consistently statistically significant correlation between *GATA3 *and *MUC1 *expression at the mRNA and protein levels by SAGE profiling (*p *= 0.002), real-time RT-PCR (*p *< 0.0001), and TMA (*p *= 0.01) in breast carcinomas.

### Identification of GATA *cis*-elements within *MUC1 *promoter

**Figure 4:**
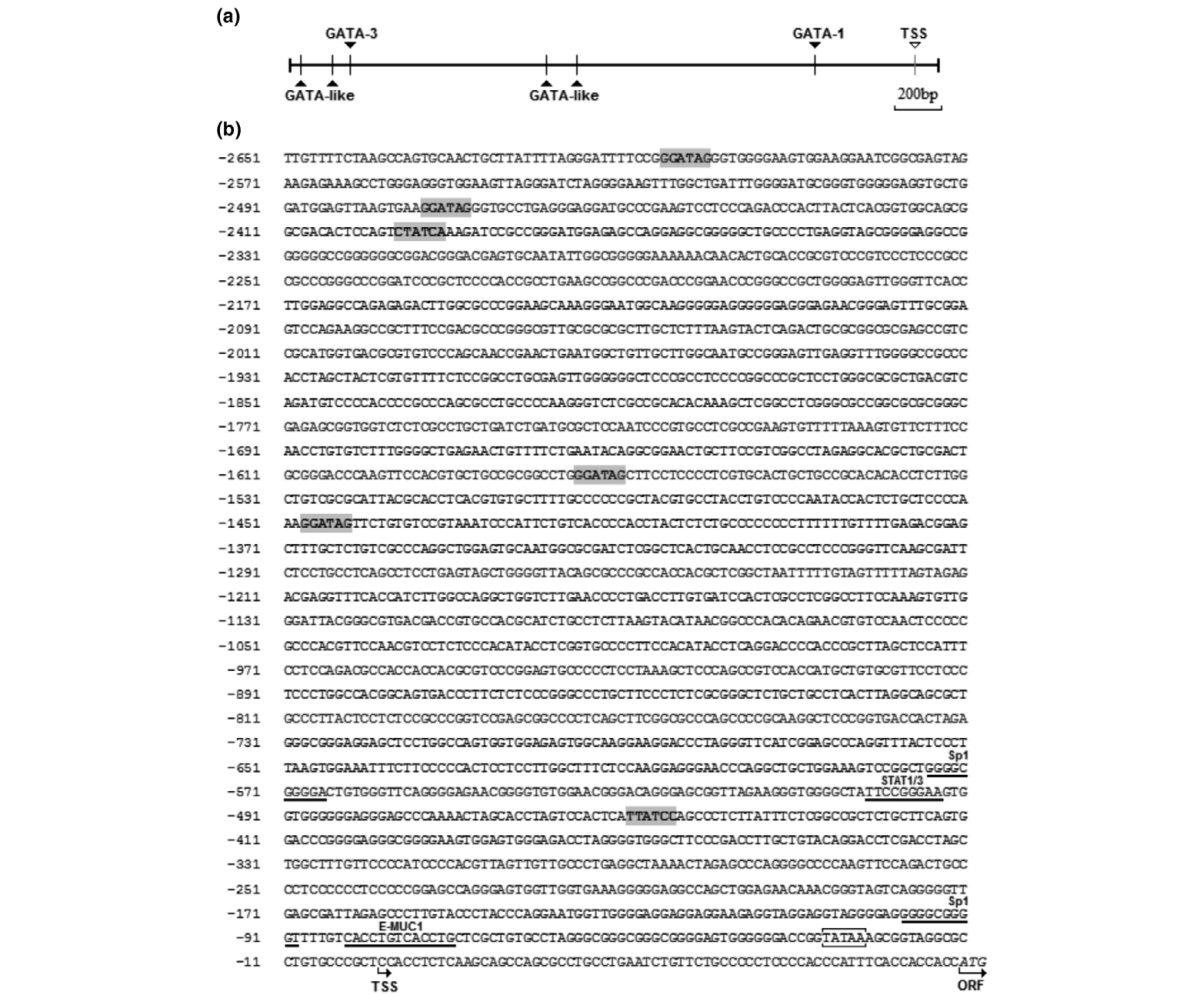
*MUC1 *promoter sequence. **(a) **GATA *cis*-elements map identified in *MUC1 *promoter region. **(b) **DNA sequence showing the TATA box (white box), the transcriptional start site +1 (TSS arrow) and the open reading frame (ORF arrow). Gray boxes indicate putative binding sites for GATA transcription factors showing the following: a GATA1 sequence (-448), a GATA3 sequence (-2395) and four GATA-like sequences (-1446, -1574, -2472, and -2604). Known regulatory elements are underlined (Sp1, STAT1/3, E-MUC1).

Analysis of the *MUC1 *promoter sequence indicated the presence of six putative GATA binding sites at -446/-451 (GATA1), -1444/-1449 (GATA-like), -1572/-1577 (GATA-like), -2398/-2393 (GATA3), -2475/-2470 (GATA-like), and -2602/-2607 (GATA-like) (Figure 4). These sites encode the conserved consensus sequence required for the binding of GATA factors. To determine whether GATA3 is capable of binding the -2398/-2393 sequence *in vivo*, we performed a chromatin immunoprecipitation (ChIP) assay with nuclear extracts obtained from the MCF7 breast cancer cell line. In brief, protein–DNA complexes were crosslinked *in vivo*, sonicated and then immunoprecipitated with anti-GATA3 monoclonal antibodies. DNA samples were obtained from three fractions: input, non-specific eluted, and immunoprecipitated. These DNA samples were subjected to PCR with two sets of primers that flanked the predicted GATA3-binding site (-2398/-2393) and the putative GATA1-binding site (-446/-451) in the *MUC1 *promoter region.

**Figure 5:**
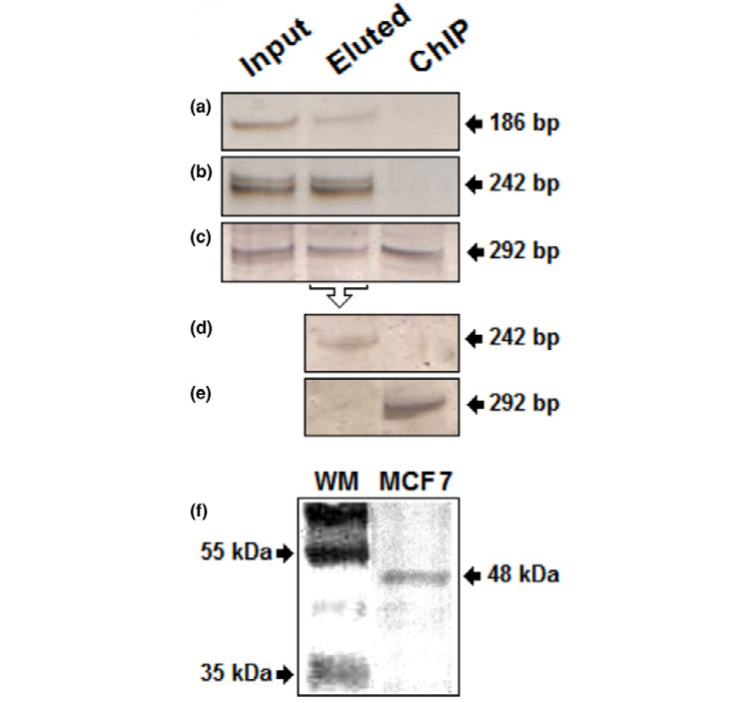
Chromatin immunoprecipitation assay of GATA3 in MCF7 breast cancer cells. Chromatin complexes were crosslinked *in vivo *with formaldehyde (see the Materials and methods section). The GATA3-associated DNA fragments were immunoprecipitated (IP) with mouse monoclonal antibody against GATA3 (HG3-31). DNA samples were isolated before IP (lane labeled 'input'), after elution the unbound proteins from the Protein A–Sepharose batch (lane labeled 'eluted') and after specific IP (lane labeled 'ChIP', for chromatin immunoprecipitation). **(a–c) **PCR was performed with (a) flanking primers amplifying a GATA1 binding site on the *MUC1 *promoter (186 bp; see Figure 2), (b) flanking primers for amplification of exonic sequences of the HLA-DQA1 gene (242 bp; negative control), and (c) flanking primers for amplification of the GATA3-binding site on the *MUC1 *promoter (292 bp) as shown in Figure 2. **(d–e) **Second ChIP (reChIP) assay using the first eluted DNA–protein complex as input sample. **(f) **Western blot analysis of GATA3 protein expression: a sample of MCF7 protein extract was run on a 10% SDS-PAGE gel and assayed for *GATA3 *expression with the monoclonal antibody used in ChIP assays (HG3-31). The expected wild-type band of 48 kDa was immunodetected. Lane WM shows an SDS-7B prestained SDS-molecular mass standard (Sigma-Aldrich).

In addition, we employed a set of primers spanning the second exon of the *HLA-DQA1 *gene (242 bp), used as negative control because there are no GATA-binding motifs in its sequence. The amplified product corresponding to the GATA1-binding site (186 bp) was detected from input DNA and non-specific GATA3-eluted DNA fractions (Figure 5a). However, this GATA1-related amplified product was not detected from the anti-GATA3-immunoprecipitated DNA fraction (Figure 5a), indicating that the GATA3 protein does not recognize the -446/-451 site from the *MUC1 *promoter region.

**Figure 6:**
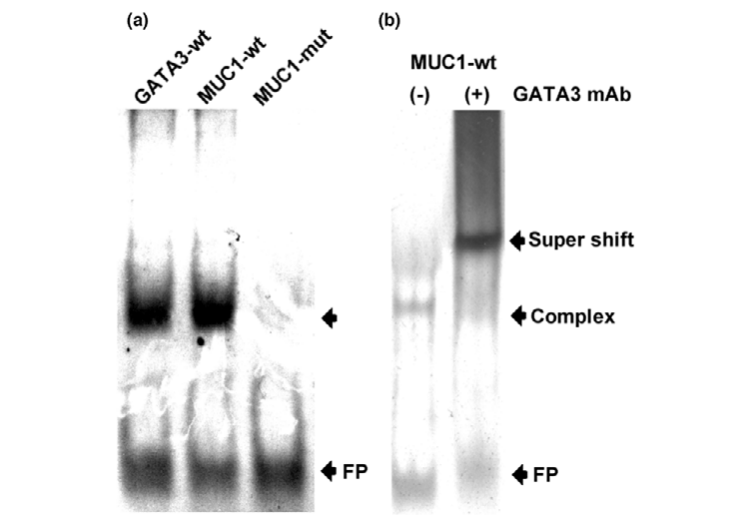
Electrophoretic mobility-shift assay employing cell nuclear extract from MCF7. **(a) **Gel shift analyses were performed with double-stranded oligonucleotides (27 bp) as indicated: positive control probe containing the GATA3-binding element (GATA3-wt), a mimetic wild-type MUC1 probe containing the predictive GATA3-binding site (MUC1-wt), and a probe sequence with predictive GATA3-binding site mutations (MUC1-mut). **(b) **The presence of GATA3 was confirmed by supershift assay. Nuclear extract prepared from MCF7 cells was preincubated with GATA3 antibody, and then incubated with the mimetic wild-type MUC1 probe containing the GATA3-binding site (MUC1-wt). Arrows to the right indicate the position of the free probe (FP), the probe–nuclear-protein complex (Complex), and the specifically retarded protein–GATA3-antibody–probe complex (Supershift).

Identical results were obtained for the *HLA-DQA1*-amplified products (negative control), demonstrating the specificity of the ChIP assay (Figure 5b). In contrast, the amplified product spanning the GATA3-binding site (292 bp in size) was detected in the input and anti-GATA3-immunoprecipitated DNA fractions (Figure 5c). These amplified products were detected only in the second immunoprecipitated DNA fraction (reChIP assay; Figure 5e). Furthermore, we employed an EMSA approach to validate the ChIP findings. Nuclear extract retarded the mobility of the *GATA3 *sequence-specific probe (positive control) and the probe encoding the putative GATA3-binding site (5'-ggcggatctttgatagactggagtgtc-3') found in the wild-type *MUC1 *promoter region (-2398/-2393) (Figure 6). In contrast, electromobility was not affected when a point mutant of the aforementioned *MUC1 *probe (T/CTTA/G instead of the wild-type T/GATA/G) was employed, indicating that no protein binding occurred (Figure 6). The presence of GATA3 in the retarded probe-protein complex was confirmed by supershift analysis (Figure 6b). The addition of anti-GATA3 antibody to the binding reaction showed a further decrease in the mobility of the probe-protein complex. Addition of the control antibody (anti-ERβ) had no effect.

Overall, these data indicate that endogenous GATA3 protein molecules from the MCF7 breast cancer cell line were able to associate with the putative GATA3-binding site of *MUC1 *promoter sequence, as demonstrated with *in vivo *and *in vitro *assays.

### *GATA3 *anti-sense assay

**Figure 7:**
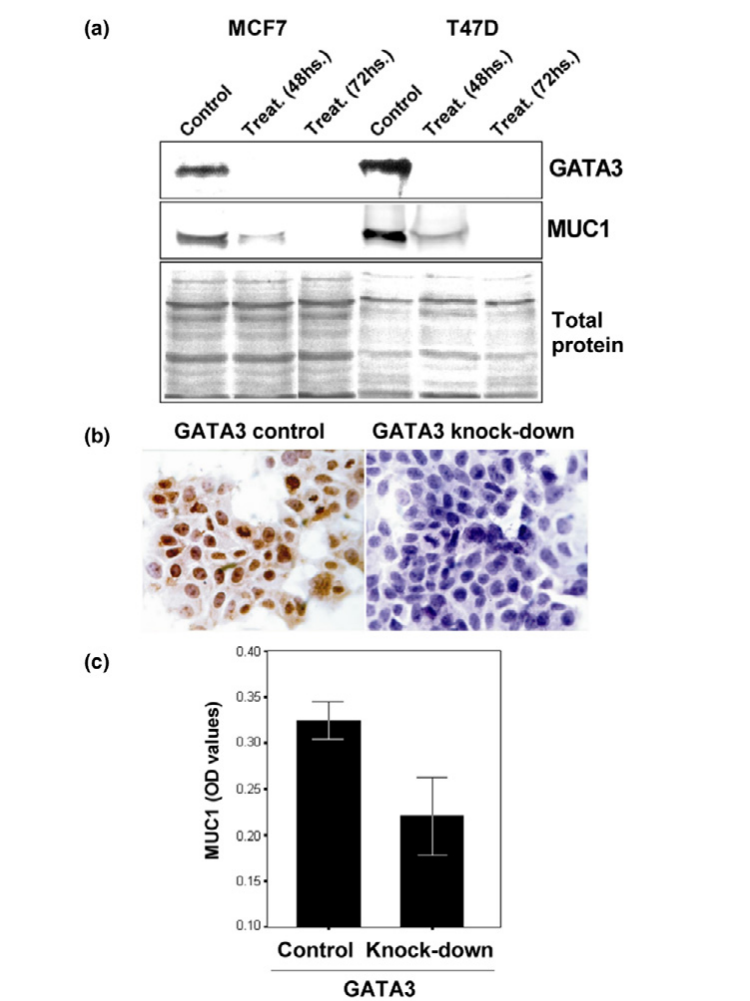
*GATA3 *knock-down analysis in MCF7 and T47D breast cancer cell lines. **(a) **Western blot analysis of GATA3 and MUC1 proteins in non-treated (control) and *GATA3 *anti-sense treated cells (48 and 72 hours) in both breast cancer cell lines. SDS-PAGE silver protein staining showed equal amounts of total protein loaded. **(b) **MCF7 immunocytochemistry of *GATA3 *anti-sense transfected cells showing no immunoreactivity to the anti-GATA3 antibody, and matching control cells show GATA3-positive nuclear localization. **(c) **Quantification of MUC1 protein expression by MCF7 culture ELISA assay. *GATA3 *anti-sense treated cells (48 hours) showed a statistically significant decrease in *MUC1 *expression compared with matched control cells (*p *= 0.001). Each experiment was performed in triplicate.

To investigate the effect of GATA3 on *MUC1 *gene expression, a *GATA3 *knock-down assay was performed with specific anti-sense oligonucleotides in MCF7 and T47D breast cancer cell lines. Cells were treated with optimal concentrations of *GATA3 *anti-sense oligonucleotides (3 μg/ml) and examined 48 and 72 hours after transfection. Immunoblotting analyses of the cells treated with MCF7 and T47D showed that *GATA3 *translation was blocked by the anti-sense oligonucleotide at both time points analyzed (namely at 48 and 72 hours after transfection; Figure 7a). Treated cells showed a significant decrease in *MUC1 *expression (Figure 7). Total protein staining showed that these results were not due to differences in protein loading. The same result was obtained in a separate experiment with a MCF7-cell ELISA assay with a different monoclonal antibody (HMFG1) against MUC1 (Figure 7b).

## Discussion

Transcriptome profile analyses demonstrated that *GATA3 *expression is strongly correlated with ERα status in breast cancer [3-9]. Recently we performed a cross-platform comparison between SAGE and DNA microarray profiles showing that the *ESR1*, *GATA3*, and *MUC1 *genes are very tightly co-expressed in breast carcinomas (Figure 1) [3]. To validate the relationship of GATA3 and MUC1 in breast tissues further, we performed mRNA expression analyses of 36 invasive breast carcinomas by real-time RT-PCR, and protein expression analysis in 263 breast tissue samples by means of immunohistochemistry on TMAs. Both analyses showed a statistical positive correlation between *GATA3 *and *MUC1 *expression at the mRNA and protein levels (*p *< 0.01; Figures 2a and 3a). Interestingly, we also detected a positive correlation between GATA3 and MUC1 protein expression when both markers were evaluated in normal breast tissue as well (*p *= 0.001).

Two recent studies from gastrointestinal epithelial cells indicated that GATA family members (GATA4 and GATA5) may have pivotal roles in the transcriptional upregulation of three mucin genes: *MUC2*, *MUC3*, and *MUC4 *[14,15]. These data led us to infer that GATA3 might have a role in the transcriptional upregulation of *MUC1 *in breast epithelium.

To determine whether GATA3 protein was actively bound to the predicted consensus *MUC1 *promoter sequence (at -2398/-2393 from the transcriptional start site (TSS)) *in vivo *and *in vitro*, we performed ChIP and gel shift analyses in breast cancer cells. The ChIP assay in MCF7 demonstrated the existence of a functional GATA3 binding site within the *MUC1 *promoter (Figure 5). The EMSA studies further validated ChIP data (Figure 6). Having found that GATA3 transcription factor efficiently recognizes a sequence motif in the distal portion of the *MUC1 *promoter, we proceeded to analyze the effects of GATA3 suppression on MUC1 protein expression in transiently transfected MCF7 cells. *GATA3 *knockdown assays led to a significant decrease in MUC1 protein expression in MCF7 breast cancer cells (Figure 7), strongly suggesting an involvement of GATA3 in the modulation of *MUC1 *expression.

The *MUC1 *promoter sequence has been analyzed previously for its ability to direct the expression of a reporter gene [19-21]. The construct (including 2.9 kb of MUC1 5'-flanking region) showed high expression levels in ZR75 cells but surprisingly low levels in MCF7 and T47D cell lines. This study showed that tissue-specific expression of a reporter gene could be obtained with only 743 bp of 5' sequences of the *MUC1 *gene, using the ZR75 cell line [19]. However, the binding of GATA3 to the promoter regions of target genes generate an open chromatin configuration that increases the accessibility to other main transcriptional regulators (perhaps STAT) in the vicinity of the TSS [30,31]. These data, together with the fact that *GATA3 *knock-down expression did not immediately abolish *MUC1 *expression, suggest that GATA3 is partly required to drive *MUC1 *expression. The finding that *GATA3 *and *MUC1 *expression were correlated in SAGE, real-time RT-PCR and TMA immunohistochemistry analyses shows statistical significance. It is also apparently a set of breast carcinomas in which there was no direct correlation between levels of *GATA3 *and *MUC1 *expression. This suggests that GATA3 may contribute to *MUC1 *upregulation in a subset of breast cancer cells (namely ERα-positive breast carcinoma).

The role of GATA3 as a transcriptional upregulator of *MUC1 *could be directly via a *cis*-acting interaction and/or indirectly via cross-talk between GATA3 and *MUC1 *co-activators such as ERα/SP1, STAT or FOG (friend-of-GATA cofactors). The transcriptional regulatory event, operating after the binding of GATA3 to the *MUC1 *promoter, remains to be determined. The *MUC1 *promoter contains several *cis*-elements specific for transcription in lymphoid cells such as STAT, MZF1, XBP1, MYB and GATA [21]. The involvement of GATA and STAT proteins in cytokine signaling is well documented in the hematopoietic and lymphoid systems [30]. Interestingly, a GATA-STAT synergy has been recently suggested as a general event that could account for cell-specific effects of transcriptional regulation [31]. The presence of these elements, which can upmodulate gene expression in lymphoid cells, are in accord with the observation that the *MUC1 *gene is expressed in activated T cells [32].

Alternatively, several putative estrogen and progesterone response elements (ERE/PRE) have been identified in the human *MUC1 *promoter by sequence analysis [33]. Zhou and colleagues [34] demonstrated that ER does not directly regulate the Muc1 promoter sequence in mice. It is possible that the *MUC1 *gene is transcriptionally regulated by non-ERE-mediated mechanisms, for example those involving ERα binding to a co-activator such as AP1 or SP1 [35]. It is interesting to note that two SP1-binding sites have been characterized as important *MUC1 *transcriptional activators in epithelial cells [19,20]. Moreover, there are studies demonstrating protein-protein interaction between ERα and GATA1 in erythroid cells [36,37]. ERα binds to the GATA1 protein at two sites, one of which is a zinc-finger domain that is highly conserved among GATA family members [36].

Therefore, a possible way of explaining *ESR1*, *GATA3*, and *MUC1 *co-expression could very probably involve the formation of a complex between the ERα and GATA3 proteins that results in the upmodulation of *MUC1 *gene expression.

It has been shown that high expression of *GATA3 *correlates with low tumor grade by *in silico *analysis of cDNA microarray data [12]. As is known, tumor grade is a good measure of breast cancer progression and differentiation status. In this study we have demonstrated a strong correlation between loss of GATA3 protein expression and more advanced stages of breast cancer progression; that is, high-grade less differentiated breast carcinomas expressed lower levels of GATA3 protein on the basis of immunohistochemical studies (*p *= 0.004). Similarly, Mehra and colleagues [38] showed that loss of *GATA3 *expression is associated with higher histological grade.

Our data agree with previously reported studies showing the role of GATA family members in the development and maintenance of a more differentiated state. Interestingly, there is emerging evidence that membrane-associated mucins (MUC1 and MUC4) contribute to the regulation of differentiation, morphogenesis, and proliferation of breast epithelial cells [17,39]. Recent data show that a fragment of the MUC1 cytoplasmic tail can be translocated to the nucleus, driving the transcriptional co-activator status of β-catenin [40]. This finding sustains the possibility that GATA3 might influence cell differentiation possibly through processes involving the upregulation of *MUC1 *gene expression.

## Conclusion

Our findings suggest that GATA3 could contribute to the transcriptional upregulation of *MUC1 *gene expression in some breast carcinomas.

## Abbreviations

bp = base pairs; ChIP = chromatin immunoprecipitation; DAB = diaminobenzidine; DTT = dithiothreitol; ELISA = enzyme-linked immunosorbent assay; EMSA = electrophoretic mobility-shift assay; ER = estrogen receptor; *ESR1 *= gene encoding estrogen receptor α; GATA3 = GATA-binding protein 3; HRP = horseradish peroxidase; IDC = invasive ductal carcinoma; MUC1 = mucin 1; PR = progesterone receptor; RT-PCR = reverse-transcriptase-mediated polymerase chain reaction; SAGE = serial analysis of gene expression; TMA = tissue microarray.

## Competing interests

The authors declare that they have no competing interests.

## Authors' contributions

MCA conceived the study idea and carried out the experimental analyses and the biostatistical/bioinformatics analysis and took part in writing the manuscript. MIN coordinated and performed pathological and tissue microarray immunohistochemical studies. AGC and MVC participated in cell culture/immunohistochemistry studies and provided practical feedback on aspects of the manuscript. CMA and AS are principal investigators and were involved in the conceptualization, design and writing of the manuscript. All authors read and approved the final manuscript.

## Supplementary Material

Additional file 1 A PDF file showing a cross-platform comparison of GATA family members expressed in breast carcinomas using SAGE, expressed sequence tag and DNA microarray databases. Click here for file

Additional file 2 A PDF file showing a principal components analysis of tissue microarray data. Click here for file
