# Supplementary material for: GATA3 protein as a MUC1 transcriptional regulator in breast cancer cells
Source: Breast Cancer Res. 2006 Nov 1;8(6):R64. doi: 10.1186/bcr1617 (PMC1797033; doi:10.1186/bcr1617)
Supplement: Additional file 1 — A PDF file showing a cross-platform comparison of GATA family members expressed in breast carcinomas using SAGE, expressed sequence tag and DNA microarray databases. [file bcr1617-S1.pdf]

Supplementary File 1: Cross-platform comparison of GATA family members expressed in breast carcinomas using SAGE, EST and DNA-microarrays databases.

Mammary gland	EST Data		SAGE Data		EST Data			SAGE Data		
	Normal	Cancer	Normal	Cancer	Normal	Cancer	P	Normal	Cancer	P
GATA1					0 / 52545	0 / 116636	--	1 / 509556	1 / 1715426	0.36
GATA2					0 / 52545	0 / 116636	--	2 / 509556	10 / 1715426	0.33
GATA3					0 / 52545	25 /116636	0.00	7 / 509556	333 / 1715426	0.00
GATA4					0 / 52545	0 / 116636	--	0 / 509556	3 / 1715426	0.32
GATA5					0 / 52545	0 / 116636	--	0 / 509556	0 / 1715426	--
GATA6					0 / 52545	1 / 116636	0.41	0 / 509556	0 / 1715426	--

Figure 1. GATA family gene expression profile in breast samples as revealed by monochromatic SAGE/cDNA virtual northern (CGAP). Spot images represent expression level of the gene. For each combination of tissue and histology, expression is computed by dividing the number of ESTs or SAGE tags representing the gene divided by the total number of ESTs or SAGE tags in all libraries with the given tissue/histology. This ratio is then multiplied by 200,000, giving the number of ESTs or SAGE tags per 200,000.

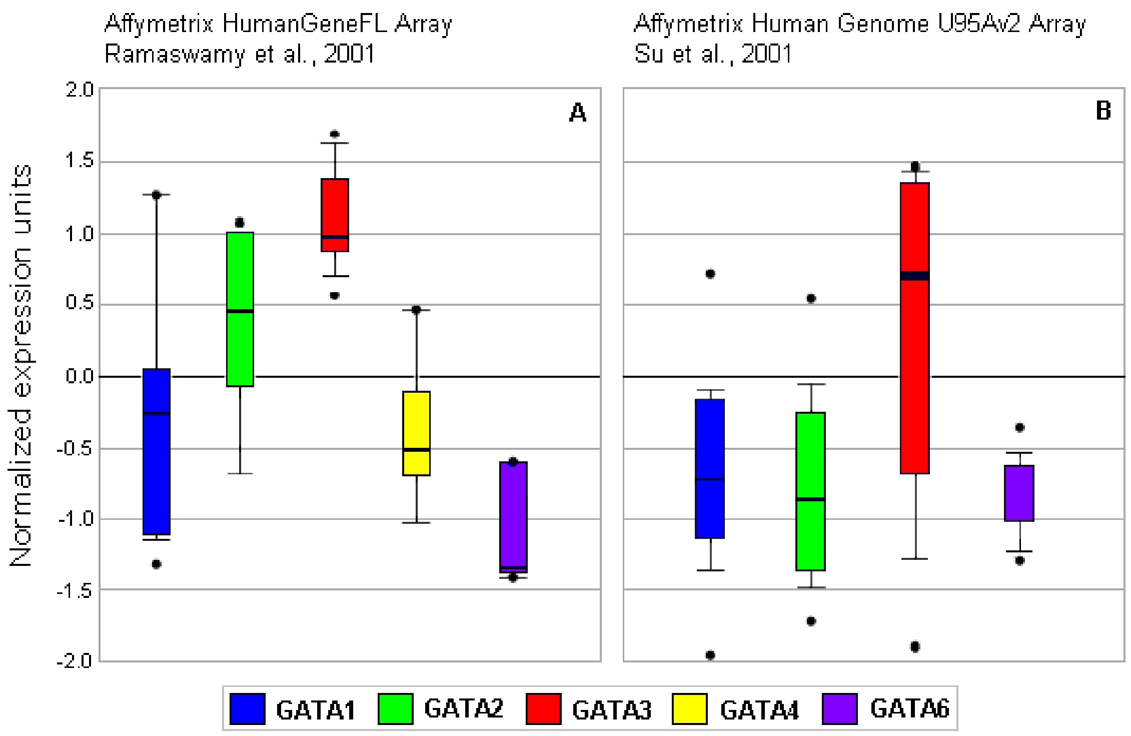

Figure 2. GATA family gene centric expression analysis as revealed by Oncomine database using breast carcinomas profiles from Ramaswamy's study (A) and Su's study (B). GATA3 gene showed high expression levels compared with the other GATA members. GATA5 transcript was not detected in breast carcinomas. GATA transcripts are represented in normalized expression units (Log2 data transformed, array median set to 0 and array SD to 1).

References of supplementary file 1

Boon K, Osorio EC, Greenhut SF, Schaefer CF, Shoemaker J, Polyak K, Morin PJ, Buetow KH, Strausberg RL, de Souza SJ, Riggins GJ. An anatomy of normal and malignant gene expression. Proc Natl Acad sci USA 2002:1-6

Rhodes DR, Yu J, Shanker K, Deshpande N, Varambally R, Ghosh D, Barrette Tm Pandey A, Chinnaiyan AM. ONCOMINE: a cancer microarray database and integrated data-mining platform. Neoplasia 2004;6:1-6.

Ramaswamy S, Tamayo P, Rifkin R, Mukherjee S, Yeang C, Angelo M, Ladd C, Reich M, Latulippe E, Mesirov JP, Poggio T, Gerald W, Loda M, Lander ES, Golub TR. Multiclass cancer diagnosis using tumor gene expression signatures. Proc Natl Acad sci USA 2001;98:15149-15154.

Su AI, Welsh JB, Sapinoso LM, Kern SG, Dimitrov P, Lapp H, Schultz PG, Powell SM, Moskaluk CA, Frierson HF, Hampton GM. Molecular Classification of Human Carcinomas by Use of Gene Expression Signatures. Cancer Res 2001;61:7388-7393.
