# Supplementary material for: GATA3 protein as a MUC1 transcriptional regulator in breast cancer cells
Source: Breast Cancer Res. 2006 Nov 1;8(6):R64. doi: 10.1186/bcr1617 (PMC1797033; doi:10.1186/bcr1617)
Supplement: Additional file 2 — A PDF file showing a principal components analysis of tissue microarray data. [file bcr1617-S2.pdf]

Supplementary File 2: Principal Component Analysis of TMA data.

Correlation Matrix

		MUC1	GATA3	ER status	PR status	Histology	Tumor grade	Lymph node
Kendall`s tau-b	MUC1							
	GATA3	**.440**						
	ER status	-.025	**.530**					
	PR status	.037	**.506**	**.700**				
	Histology	-.073	-.009	-.095	-.004			
	Tumor grade	-.041	**-.490**	**-.668**	**-.528**	.108		
	Lymph node	**.462**	.099	.063	-.031	.139	.061	
p value	MUC1							
	GATA3	.011						
	ER status	.385	.000					
	PR status	.334	.001	.000				
	Histology	.197	.456	.133	.480			
	Tumor grade	.318	.002	.000	.000	.103		
	Lymph node	.006	.124	.229	.357	.052	.238	

Color code:
Statistical significant positive correlations
Statistical significant negative correlations

Total Variance Explained

Component	Initial Eigenvalues			Extraction Sums of Squared Loadings			Rotation Sums of Squared Loadings		
	Total	% of Variance	Cumulative %	Total	% of Variance	Cumulative %	Total	% of Variance	Cumulative %
1	2.042	29.165	29.165	2.042	29.165	29.165	2.005	28.643	28.643
2	1.293	18.476	47.641	1.293	18.476	47.641	1.291	18.441	47.084
3	1.064	15.195	62.835	1.064	15.195	62.835	1.103	15.752	62.835
4	.790	11.284	74.119						
5	.708	10.110	84.230						
6	.683	9.756	93.986						
7	.421	6.014	100.000						

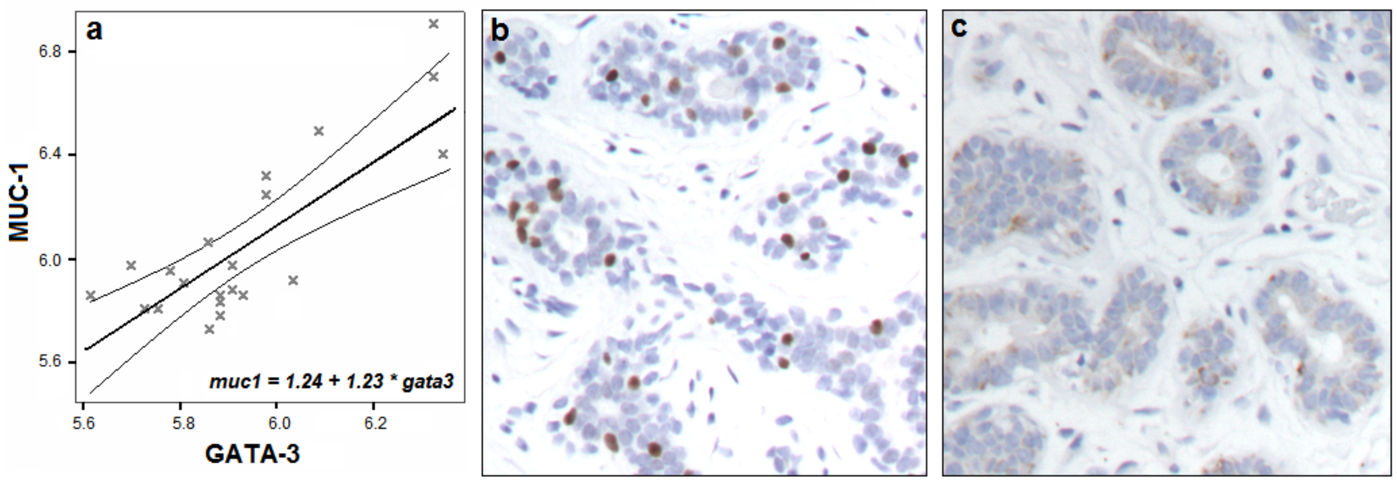

Supplementary figure. GATA3 and MUC1 immunohistochemical staining in normal breast samples.

a) Linear regression analysis between both markers with 95% mean prediction interval (r=0.78, p=0.001);

b) GATA3 nuclear positive immunostaining; **c**) MUC1 apical cytoplasmatic immunostaining.
